# A Fast and Robust Deep Convolutional Neural Networks for Complex Human Activity Recognition Using Smartphone

**DOI:** 10.3390/s19173731

**Published:** 2019-08-29

**Authors:** Wen Qi, Hang Su, Chenguang Yang, Giancarlo Ferrigno, Elena De Momi, Andrea Aliverti

**Affiliations:** 1 Dipartimento di Elettronica, Informazione e Bioingegneria, Politecnico di Milano, 20133 Milano, Italy; 2 Bristol Robotics Laboratory, University of the West of England, Bristol BS16 1QY, UK

**Keywords:** human activity recognition, convolutional neural network, data compression

## Abstract

As a significant role in healthcare and sports applications, human activity recognition (HAR) techniques are capable of monitoring humans’ daily behavior. It has spurred the demand for intelligent sensors and has been giving rise to the explosive growth of wearable and mobile devices. They provide the most availability of human activity data (big data). Powerful algorithms are required to analyze these heterogeneous and high-dimension streaming data efficiently. This paper proposes a novel fast and robust deep convolutional neural network structure (FR-DCNN) for human activity recognition (HAR) using a smartphone. It enhances the effectiveness and extends the information of the collected raw data from the inertial measurement unit (IMU) sensors by integrating a series of signal processing algorithms and a signal selection module. It enables a fast computational method for building the DCNN classifier by adding a data compression module. Experimental results on the sampled 12 complex activities dataset show that the proposed FR-DCNN model is the best method for fast computation and high accuracy recognition. The FR-DCNN model only needs 0.0029 s to predict activity in an online way with 95.27% accuracy. Meanwhile, it only takes 88 s (average) to establish the DCNN classifier on the compressed dataset with less precision loss 94.18%.

## 1. Introduction

With the widespread usage of portable and wearable smart devices, human activity recognition (HAR) becomes an active and comfortable research field. Various device-embedded sensors gather human motion data using the method proposed by the authors of [1]. Continuous HAR systems are developed as part of a framework to monitor long-term human behaviors, such as ambient assisted the living, sports injury detection, and surveillance [2]. A HAR system is expected to report on people’s daily activities outside a hospital setting becomes an essential tool for healthcare interventions evaluation and clinical decision-making [3]. In a home setting, the wearable sensor technology can potentially facilitate many applications such as rehabilitation instruction, motion evaluation, activity reminder, and fall detection [4]. As an increasingly important role, HAR improves life quality and promoting health at an individual. In assisted living systems, HAR helps bridge the gap between the low-level sensor and the high-level human-centric applications. Among the various available sensing components, the smartphone is widely used due to its low intrusiveness, convenience, and high adherence. Overall, monitoring daily human activity provides a significant reference for healthcare, such as prevent or delay diabetes, blood glucose control, heart failure, and cardiovascular disease [5]. Smartphones offer too much convenient for monitoring human’s physical and physiological parameters by embedding many suitable sensors, such as the inertial measurement unit (IMU), oximeter, thermometer, and GPS [6]. Currently, these kinds of the smartphone are omnipresent in society.

To HAR performance enhancement, some of the pioneering classification approaches using the IMU (smartphone) sensors had been studied for many years. Moreover, it will be researched extensively. A Robust Least Squares Twin Support Vector Machine (RLS-TWSVM) algorithm [7] was used for addressing the presence of noise between related activity classes along with the high computational time of the HAR system. By extracting 248 features from the integrated sensors in smartphones, a HAR system based on Support Vector Machine (SVM) [8,9] was written on Windows and Android platforms and operated in real-time could identify six actions, i.e., standing, sitting, walking, lying, and walking up and downstairs. To achieve the real-time HAR, a time series segmentation method based on k-Nearest Neighbor (k-NN) [10] and Artificial Neural Network (ANN) [11,12,13] was described by collecting data from a single triaxial accelerometer from a smartphone. Primarily, it must assume that each meaningful segment corresponds to one fundamental period of motion. Unfortunately, the above approaches were implemented in the controlled lab environment, and the smartphone was fixed on body position. The embedded accelerometer and gyroscope in a smartwatch had been especially used for HAR [14]. The comparison results of six classes classification by using accelerometer, gyroscope, and combined sensors proved that the combined sensors are useful. However, research for using multiple body-worn IMU sensors in a free-living situation to recognize a wide range of activities became the most challenging research. Fullerton et al. [15] tried to recognize activity through adopting multiple accelerometers in a free-living environment. The comparison results shew the k-NN classifier reported the highest recognition accuracy (97.6%) with mean and standard deviation features of unfiltered data, while the decision tree (DT) [16] method demonstrated the lowest computing time. The k-NN algorithm [11] was evaluated as the fastest method to build a classifier for classifying six activities (i.e., walking, sitting, standing, and going upstairs and downstairs) by comparing with other four approaches such as logistic regression (LR), ANN, SVM, and J48 DT.

With the growing dimension of the inputs, the number of selected nearest neighbors k in k-NN method becomes difficult to search. A Particle Swarm Optimization (PSO)-based k-NN algorithm [17] was employed for finding the optimal value of k and was successfully applied for recognizing 19 activities sampled from three body positions. Some complex activities were considered to be detected, such as writing, typing, talking, and drinking coffee, by wearing a wrist-worn motion sensor and carrying smartphone together [18]. Although the k-NN method always got better performance than other traditional ML algorithms, it needed to extract more features for building the classifier [19]. To solve this problem, a clustered k-NN algorithm [20] was designed to eliminate the computational complexity of the previous k-NN method by creating clusters, for example, creating smaller training sets for each activity and performing the classification processing based on these reduced sets. By selecting in consultation with the first-responder from inertial data, the Gradient Boosted Trees (GBT) [21] could be applied to recognizing 17 activities. Moreover, it was proved to get better performance than k-NN and SVM algorithms [22]. The Levenberg–Marquardt (LM)-based ANN [23] algorithms have better HAR performance (by 92.81% accuracy) though comparing with Quick Propagation (QP) and Batch Back Propagation (BBP) based ANN methods on Massachusetts Institute of Technology (MIT) smart home dataset. The comparison results illustrated that the Naive Bayes (NB) classifier acquired the highest F-Measure value using both a‘ccelerometer and gyroscope. However, the data collection was performed in a supervised environment, and the classification should be optimistic in real-world. To enhance the efficiency of the extracted features, Hassan et al. established a series of operational mechanisms. The features, i.e., mean, median, and autoregressive coefficients, were extracted from the triaxial angular velocity and linear acceleration signals. Further, a kernel principal component analysis (KPCA) and linear discriminant analysis (LDA) were used to make the features more robust. Finally, they adopted the Deep Belief Network (DBN) algorithms to build the activity classifier. The high performance of this approach was proved by comparing with SVM and ANN on the same dataset.

Unfortunately, all of the above traditional ML algorithms depended on a features extraction (FE) module. In other words, the performance of the designed FE algorithms decided the recognition rate and computational speed of the classifier [24], for example, k-NN, SVM, DT, and ANN methods [25] only worked well under the premise of extracting perfect features from the collected signals. Meanwhile, this HAR architecture was designed based on ’shallow’ layer features and obtain an optimum performance dependent on the set task [26]. The most challenging part of the mobile and wearable sensor-based HAR pipeline was the extraction of relevant features [27]. It influenced the classification performance, computation time, and complexity. However, with the growing of the influx of multimodal and high dimensional sensor data, the current HAR system based on the above mentioned ML algorithms were incapable of handling complex activities [28]. Most of them relied on handcrafted features. Besides, for obtaining a high classification accuracy, most of the evaluation of the experiment was limited in a controlled area for collecting data. Moreover, the complexity of human motions brings more difficulties for accuracy enhancement [29]. Many commercial frameworks were implemented for performing these problems [30]. An efficient group-based context-aware method was proposed for exploiting hierarchical group-based scheme to improve the classification efficiency [31].

The emergence of deep learning (DL), artificial intelligence (AL), and computation powers techniques [32,33,34] skipped the step to extract features manually. The DL method was being adopted for automatic feature learning in diverse fields like healthcare, image classification, and recently, for complex HAR in mobile and wearable sensors [35]. Recently, a large number of DL algorithms had been successfully implemented in HAR by selecting features spectrum automatically. An efficient and effective HAR model based on deep convolutional neural network (CNN) [36] was proposed to exploit accelerometer, gyroscope, and the inherent characteristics of activities. In this paper, Ronao et al. also provided a way to automatically and data-adaptively extract robust features from raw data with the 1D time series signals. The transformed frequency spectrum was implemented on three public datasets based on a designed DCNN model [37] with four CNN modules. By comparing with another feature-based method, the proposed DCNN approach acquired the best performance on all of the datasets. A DCNN model with three CNN layers and two maximum pooling layers [38] was successfully identified eight activities. Notably, this model overcame the 8-layer Deep Belief Network (DBN) for recognizing each behavior. Due to the scarcity of labeled training data and the poor classification accuracy of existing recognition methods, a deep activity recognition system based on Gaussian-binary restricted Boltzmann machines (GRBMs) [39] was proposed. A bidirectional Long short-terms memory (Bi-LSTM) structure was proposed [40] for identifying six daily activities. It used both acceleration and angular velocity signals to build the Bi-LSTM classifier. Even though it obtained 93.79% classification accuracy, the types of activity were so simple, which also can be identified well by many traditional ML algorithms. The possible rotational interference present in the raw signals decreased the recognition rate of HAR [1]. To solve this problem, a robust one-dimensional (1D) Convolutional Neural Network (CNN)-based method was presented by utilizing vector magnitude accelerometer data. The deep CNN structure was also suitable for identifying the data coming from the multichannel time series [41]. Although the proposed deep CNN model obtained the best classification performance by comparing with other ML methods, like k-NN, SVM, and means and variance (MV), the less number of subjects in the selected dataset were not enough to measure the claims. For fast recognition, a new deep recurrent neural network (DRNN) was proposed [42]. By using both acceleration and angular velocity, a presented DCNN model obtained a high recognition rate (94.79%). Moreover, it gets a higher accuracy (95.75%)by adding the frequency spectrum information. By comparing deep feedforward neural network (DNN) [43], CNN and RNN on three representative datasets, CNN structure was proved as the best algorithm [44]. To achieve a real-time classification for low-power wearable devices, a deep temporal convolution network [24] was designed and applied on the frequency spectrum calculating by short-time, fast Fourier-transform (STFT). Capturing these temporal dynamics during human motion period is fundamental for successful HAR. A deep generic framework based on CNN and RNN (LSTM) units were implemented on multimodal wearable sensors [45]. It was independent of the knowledge in designing features and could perform sensor fusion naturally.

Nweke et al. [2] summarized and divided the state-of-the-art DL methods for HAR using a smartphone and wearable sensor into five-folds, i.e., restricted Boltzmann machine, autoencoder, sparse coding, DCNN, and DRNN. However, there still had many challenges for HAR. For example, to implement the DL algorithms on mobile and wearable devices was energy-consuming, and it was difficult to collect large sensor datasets. The most difficult challenge of HAR was how to classify the no or minimal labeled data. Hence, exploring DL structures with fast computation and high precision is a hot topic [2]. A transfer learning motivated CNN-based HAR framework was proposed (called Heterogeneous DCNN) [46], which could automatically adapt and learn the model in new unlabeled data. The method was evaluated on real-world data and achieved high accuracy, while these conclusion needed to be proved on more data sampled from the non-stationary environment. Most recently, many contributions were explored the unsupervised HAR. The Molecular Complex Detection method (MCODE for short) [47] was utilized on recognizing daily living activities (e.g., walking, race walking and running), and basketball playing (e.g., standing, bouncing or passing the ball, free throw, and moving with ball). By comparing with other clustering algorithms, like Gaussian Mixture Model (GMM) [48] with Expectation Maximization, hierarchical clustering method (HC) [49], two centroid-based clustering methods (k-means [50] and k-medoids [51]) and a graph-based clustering method (spectral clustering) [52], the MCODE methods acquired the highest performance of classification.

By summarizing the previous contributions of HAR, such as methods, platform, systems, and algorithms, monitoring human activity by using the signals acquired from IMU sensors in the field is complicated by the following three difficulties.

- Although it can reduce the computational complexity of data storage and transfer for the onboard implementation of DL algorithms on smartphone and wearable devices, this technique is hampered by data acquisition and memory constrained. Therefore, exploring optimal compression methods and adopting mobile phone enabled GPU to minimize computation time is highly needed.
- The signals processing and dimensionality reduction are two significant aspects of HAR process for enhancing the recognition rate. The acquired new signals with low dimensional data minimize computational complexity, especially in mobile devices with limited computation powers and memory.
- Human activity is too complex to be recognized because it is easy to be affected by the user’s habit, age, physical status, and even wearable devices. For example, there are many transitions between two activities, such as from sitting to standing [53]. Thus, to explore an algorithm to identify the transfer motions and other complex activities becomes more popular.

This paper proposes a new fast and robust deep convolutional neural networks (FR-DCNN) architecture to identify 12 complex human activities collected from a smartphone. It includes five exercises (i.e., walking, jogging, jumping, and go upstairs and go downstairs), six postures (i.e., sitting, standing, lying to the left and right side, and lying supine and prone), and a series of transitions, (e.g., from standing to sitting). This study aims to train and apply the FR-DCNN model, which can accurately recognize the 12 activities and fast establish the DCNN classifier. To achieve the claims, a data compression module is designed to remove the similarity training segments for saving the computational time of building the DCNN classifier. A signal processing model is established by adopting several denoising and matrix rotation methods for overcoming the defects of white noises and rotational interference. Moreover, a new DCNN structure is designed with dropout layer to reduce the training time.

The outline of this article is as follows. Section 2 describes the wireless communications system for data collection and explains the details of the sampled signals. Subsequently, it introduces the architecture of the proposed FR-DCNN model. Section 3 compares the performance (e.g., classification accuracy and computational time) among FR-DCNN and the state-of-the-art algorithms. Finally, Section 4 summarizes the works presented in this paper and presents the future works.

## 2. Materials and Methods

### 2.1. Data Preparation

**Figure 1:**
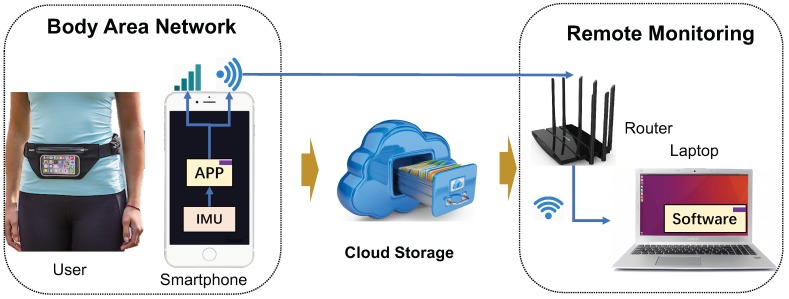
The schematic diagram of data collection and wireless communication between the user carrying the mobile device and the data analyzing unit (laptop). The activities can be identified on both smartphone and PC platform. The cloud storage can save the collected signals for future analysis.

For collected the mentioned activities in Section 1, a wireless communication network [54] (shown in Figure 1) was built among the user, the smart devices (smartphone), and the data analyzing unit (laptop). The users were instructed to record the 12 activities by carrying the smartphones (iPhone 6s) fixed on the waist. It included five types of dynamical exercises (i.e., walking, jogging, jumping, and going upstairs and downstairs), six static postures (namely, sitting, standing, lying to the left and right side, and lying supine and prone), and a series of transitions, (e.g., from standing to sitting). All three types of IMU sensors were used for data gathering: accelerometer, gyroscope, and magnetometer. We acquired the raw data by installing the MATLAB app in the mobile phone and sent them to the cloud storage through a wireless connection (cellular network or Wi-Fi). The proposed HAR algorithm was run in the smartphone and the PC for identifying the collected signal segments in real-time. Finally, the whole wireless communication system can monitor the subject’s activity both on the smartphone and PC. The raw signals had nine dimensions because each sensor provided three-axis data (i.e., *x*, *y*, and *z*). They were recorded at 50Hz frequency (i.e., 50 samples per second) along with the activity labels per given timestamp (hand-labeled by the observer).

**Figure 2:**
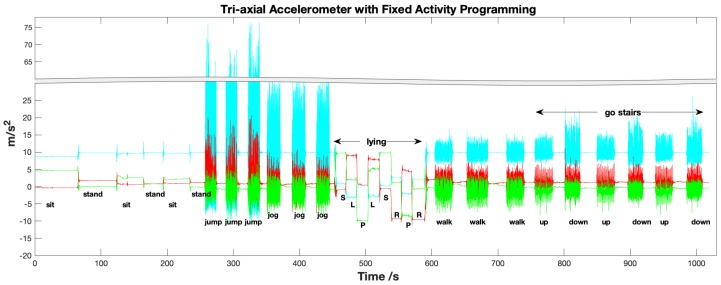
The schematic diagram of labeled 12 activities on the collected 3-axis acceleration signals.

Twenty subjects (ten males and ten females, age range from 21 to 30) were asked to do the 12 activities with fixed order and experimental protocol, as it was described in Figure 2. To satisfy the demands of building a DCNN classifier for applying on more users, it needs to collect a large sampling dataset. Hence, the subjects are asked to do each activity for about one minute. Usually, a total of 25 to 30 min data would be obtained from one subject with the fixed order in Figure 2. Moreover, it would get more than $5\times 10^{4}$ samples with 50Hz sample frequency. The testing started from three times sitting and standing postures. Then, the subjects would be asked to repeat the jumping and jogging three times. To avoid making them too tired, they could stand during those strenuous exercises period. The four kinds of lying postures (supine, prone, and lying to the left and right side) had been done twice with several different transition activities. For example, one kind of transfer activity was labeled as lying supine to prone. The rest dynamic exercises (i.e., walking and going upstairs and downstairs) were also repeated three times. The ’standing’ motion could be collected many times because it helps the subjects to get a break. For instance, the user could get relax during the walking testing period.

Finally, it could acquire ~3 min data of the following activities, namely sitting, jumping, jogging, walking, go up and downstairs. It would collect ~2 min of data of the four types of lying positions. Moreover, much more extended times of standing. The transition process presented a stage when people transferred from one motion state to another. For example, a lying transition means that the user changes their position from lying supine to lying prone.

### 2.2. The Proposed Method

**Figure 3:**
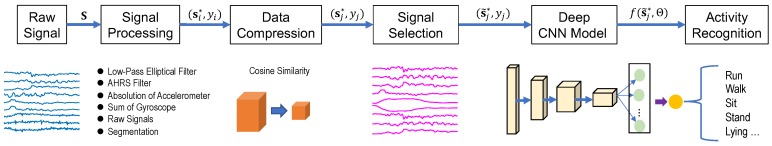
The structure and data stream of the fast and robust deep convolutional neural networks (FR-DCNN) for complex human activity recognition (HAR). The signal processing module is to extend the dimension of raw signals and label the divided segments. The data compression module aims to remove similar components for fast computation. The signal selection module is to choose the useful signals for high-accuracy classification. The new designed DCNN algorithm is to build the DL classifier for the next step—activity recognition.

To achieve online (or real-time) monitoring human activity [55,56,57], identifying the motions on each segment is required. According to the description of collecting dataset in Section 2.1, many similar components might be produced to causes the computational efficiency for training the DL classifier. Meanwhile, several noises are coming from human movement and the direction change of smartphone, which decrease the recognition rate of HAR. To improve the computational speed and classification accuracy, we propose a new fast and robust structure based on deep convolutional neural networks (DCNN), called FR-DCNN model. Figure 3 introduces the structure of the FR-DCNN with data stream procedure. The raw signals are collected from an embed IMU sensor, including a tri-axis accelerometer, a tri-axis gyroscope, and a tri-axis magnetometer. Hence, the raw signals have nine dimensions (9D), namely $S^{9}$. It can be processed four signal processing methods, i.e., low-pass elliptical filter, attitude and heading reference system algorithm (AHRS filter), absolution of accelerometer, and the sum of angular velocity. As the next step is to remove the similar components for fast computation, the chosen method must be able to avoid deleting too many segments in a low-dimensions level. For achieving this aim, we add the 9D raw signals into the processed signals through the signal processing module. Hence, the processed signals have 19 dimensions, i.e., $s_{i}^{*},i=1,2,\cdots ,N,s^{*}\in R^{19}$. Subsequently, the 19D signals will be separated into several segments by a fixed detection length with the slide window mechanism. Moreover, they will be labeled by the marked classes for building the classifier in the future, namely $(s_{i}^{*},y_{i}),y\in \lambda$. Where Λ is a discrete class label in the classification problems. Due to the extended dataset increases the dimensions of the raw signals set, it can leave the useful information after deleting the similar segments by the data compression module. This module is designed by computing the cosine similarity between two segments with the same label. For achieve fast computation and high accuracy classification, it is better to select some of the components from the 19D signals to build the classifier. The selected inputs $\left({\tilde{s}}_{j}^{*},y_{j}\right),j=1,2,\cdots ,M,{\tilde{s}}^{*}\in R^{m}$, where M≤N and m≤19, is acquired through the signal selection module. The labeled and selected segments are used for establishing the DCNN classifier. Finally, the DCNN classifier can be applied on activity recognition in an online way.

#### 2.2.1. Signal Processing

As described in Section 2.2, the embedded IMU sensors in a mobile phone consists of an accelerometer, a gyroscope, and a magnetometer. They can provide 9D original signals, namely, proper acceleration $S_{\alpha }$, angular velocity $S_{\beta }$, and magnetism $S_{\gamma }$—the direction, strength, or relative change of a magnetic field at a particular location, respectively. When people does different activities by carrying the smartphone on a fixed body position, the values of $S=\{S_{\alpha },S_{\beta },S_{\gamma }\}$ will change. So, it can classify human activity by analyzing the collected dynamic signal. Unfortunately, the performance of the established HAR classifier will be affected by several kinds of noises and interference. To improve the identification ability, the proposed FR-DCNN model adopts a series of signal processing algorithms to extend the dimensions of raw signals. That can expand the information of raw signals. Meanwhile, several filters are adopted for denoising. There are four types of signal processing algorithms are chosen in this article. The third-order zero phases Low-Pass Elliptic Filter (LPEF) [58] is implemented to divide the raw acceleration into a gravity and the linear acceleration $S_{\alpha^{1}}$. The LPEF filter removes the abrupt changes and high-frequency components by the gain function as follows.

(1) $$G_{n}\left(f\right)=\frac{1}{\sqrt{1+\varepsilon^{2}C_{n}^{2}\left(\tau ,f/f_{c}\right)}}$$

where the cutoff frequency $f_{c}$ is round between 0.3 Hz and 20 Hz, ε is the ripple factor, and τ is the selectivity element. $C_{n}$ is the *n*th-order Chebyshev rational function [59].

The meaning of the tri-axis accelerometer will change along with the changing of the direction of the smartphone. Hence, it is difficult to identify the four positions of lying mentioned in Section 1. We choose two methods to solve this problem. First, the absolution of acceleration $S_{\alpha^{2}}=\left|S_{\alpha }\right|$ will be used for reduce the direction change of the smartphone. Second, the attitude and heading reference system algorithm (AHRS filter) [60] help to fix the three axes of both acceleration and magnetism by calculating the orientation. The obtained 3-axis orientation $S_{\delta }$ is computed according to the three raw sensors—$S_{\alpha }$, $S_{\beta }$, and $S_{\gamma }\}$—by Eqution (2).

(2) $$\left[\begin{array}{c} S_{\delta_{t}}\\ S_{\alpha_{t}}\\ S_{\beta_{t}}\\ S_{\gamma_{t}} \end{array}\right]=F_{t}\left[\begin{array}{c} S_{\delta_{t-1}}\\ S_{\alpha_{t-1}}\\ S_{\beta_{t-1}}\\ S_{\gamma_{t-1}} \end{array}\right]+\omega_{t}$$

where $\omega_{t}$ is 12-by-1 additive noise vector and $F_{t}$ is the state transition model. The orientation components also increase the accuracy of identifying the two similar activities, for example, go upstairs and downstairs. Notably, the orientation is proved to divide the four lying positions, because it will not be affected by the detection change of the smartphone. Finally, since the angular velocities are shown to provide more information for identifying the dynamical activities, especially the most similar motions (e.g., going upstairs and walking). We add an information of the sum of the three angular velocities $S_{\beta^{1}}=S_{\beta_{x}}+S_{\beta_{y}}+S_{\beta_{z}}$ in $s^{*}$ to solve this problem.

Finally, the raw 9D signals are extended into 19 dimensions based on the above transitions.

(3) $$S=\left[S_{\alpha },S_{\beta },S_{\gamma }\right]\Rightarrow S^{*}=\left[S_{\alpha },S_{\beta },S_{\gamma },S_{\alpha^{1}},S_{\alpha^{2}},S_{\beta^{1}},S_{\delta }\right]$$

where the $S_{\alpha },S_{\beta }$, and $S_{\gamma }$ are the three original signals. $S_{\alpha^{1}}$ and $S_{\alpha^{2}}$ are the linear acceleration and the absolution of acceleration, respectively. $S_{\beta^{1}}$ is the sum of the angular velocities. $S_{\delta }$ is the orientation.

Afterward, it will be divided into several labeled segments by a fixed detection length $L_{d}$ with the slide window mechanism [61], namely, $(s_{i}^{*},y_{i}),i=1,2,\cdots ,N$. Where y∈Λ is a discrete class label sequence.

#### 2.2.2. Data Compression

For achieving the fast computation on building the DCNN classifier, we design a data compression module to delete the similar segments in $s_{i}^{*},i=1,2,\cdots ,N$ with the same label. The obtained 19D segments sequence $s_{i}^{*},i=1,2,\cdots ,N$ have many similar (even same) components in the same labeled dataset. This is the main reason for causing time-consuming for training the DL classifier. Meanwhile, it will occupy more storage. The method get some idea from seeker optimization algorithm (SoA) which is based on the concept of simulating the act of the intelligent of humans searching with experience, memory, and uncertainty [62,63]. When the start point, search direction, search radius, and trust degree are given, every seeker moves to a new position (next solution) by the cognitive or social learning, and uncertainty reasoning [64]. The SoA algorithm provides new idea for solving the problem of time-consuming and energy-consuming on DL programming [65,66]. To solve the problem, the cosine similarity method [67] is adopted to compute the similarity degree between two segments in the same labeled dataset. The procedure of this method is to measure the similarity coefficient ρ by calculating the cosine of the angle between two non-zero vectors ($s_{a}^{*}$ and $s_{b}^{*}$) of an inner product space as

(4) $$\rho =\frac{s_{a}^{*}\cdot s_{b}^{*}}{∥s_{a}^{*}∥∥s_{b}^{*}∥}=\frac{\sum_{k=1}^{K}s_{a_{k}}^{*}s_{b_{k}}^{*}}{\sqrt{\sum_{k=1}^{K}{\left(s_{a_{k}}^{*}\right)}^{2}}\sqrt{\sum_{k=1}^{K}{\left(s_{b_{k}}^{*}\right)}^{2}}}$$

In this article, when ρ≤0.99, we regard the two segments are similar and delete one of them. To avoid removing too much segment from the same labeled dataset, we define the coefficient ζ in 5 to control the compression process.

(5) $$L^{*}=\zeta \times L$$

When ζ=0.5, it means there are 50% segments with the same labeled dataset will be removed.

#### 2.2.3. Deep Convolutional Neural Networks

**Figure 4:**
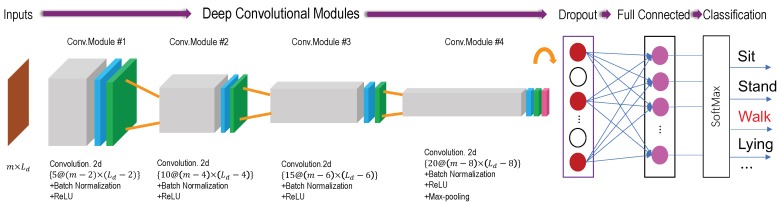
The structure of Deep Convolutional Neural Networks.

After acquired the training dataset, it can build the classifier for HAR. The procedure of training a DCNN classifier on $\left({\tilde{s}}_{j}^{*},y_{j}\right),j=1,2,\cdots ,M,{\tilde{s}}^{*}\in R^{m}$ can be regard as a supervised learning problem. The designed DCNN structure consists of four deep convolutional modules, a dropout layer [68], a full connection layer, and a classification module. The first three deep convolutional modules consist of a 2D convolution layer [69], a batch normalization layer [70] and a rectified linear units (ReLU) [71]. The last deep convolutional module add a max-pooling layer [72]. Figure 4 shows the 2D DCNN architecture of our proposed method.

The details of the DCNN frame can be described as follows.

- Inputs: A matrix with *m* dimensions and fixed time-length, namely ${\tilde{s}}_{m\times L_{d}}^{*}$. Specifically, we constructed four kinds of inputs for comparison experiments, i.e., ${\tilde{s}}_{3\times L_{d}}^{*}=\left[s_{\alpha }\right]$, ${\tilde{s}}_{6\times L_{d}}^{*}=\left[s_{\alpha },s_{\beta }\right]$, ${\tilde{s}}_{9\times L_{d}}^{*}=\left[s_{\alpha },s_{\beta },s_{\gamma }\right]$ and ${\tilde{s}}_{9\times L_{d}}^{*}=\left[s_{\alpha^{1}},s_{\alpha^{2}},s_{\beta^{1}},s_{\delta_{Y,Z}}\right]$. Where $s_{\delta_{Y,Z}}$ represent the Y and Z axis of orientation signals. Figure 4 ’inputs’ shows the 3 s input vector of a given training data size, namely $L_{d}=150$ with 50 Hz sample frequency.
- Deep Convolution Modules: Four deep convolutional modules are designed in DCNN model. The first three modules (Conv.Module #1 to Conv.Module #3), consist of a 2D CNN layers, a batch normalization (BN) layer and a Rectified Linear Unit (ReLU) activation function. The last convolution module Conv.Module #4 add a max-pooling layer. The convolution operations were performed using the four window sizes, 5, 10, 15 and 20. The size of yielded feature map are $\left(m-2\right)\times \left(L_{d}-2\right)$, $\left(m-4\right)\times \left(L_{d}-4\right)$, $\left(m-6\right)\times \left(L_{d}-6\right)$, and $\left(m-8\right)\times \left(L_{d}-8\right)$. The convolution operations were performed using the window size 3×3. The BN layer is adopted for allowing each layer of the CNN network to learn by itself a little bit more independently of other layers. The ReLU layer aims to solve the vanishing gradient and exploding gradient problems [73].
- Max-pooling: The max-pooling was performed to select the largest feature value finally. The max-pooled result acquired from the last layer of Conv.Module #4 reshape to create a feature vector for the input matrix.
- Dropout: The convolved and max-pooled feature vectors usually too large to cause overfitting problem. This phenomenon will decrease the classification accuracy. The dropout layer is applied for avoiding overfitting and reducing the training time. The percentage of the dropout is set to 0.5 in our evaluation experiment to be explained later.
- Output: The SoftMax layer was placed as an output layer of the fully-connected layer as shown in Figure 4. It classify the activities by computing the probability of each input of the node in the softmax layer, like sitting, standing, walking and lying. The highest probability is then determined as the predicted (or recognized) activity. Finally, the activity label is outputted to the final node (in red).

## 3. Results and Discussion

**Figure 5:**
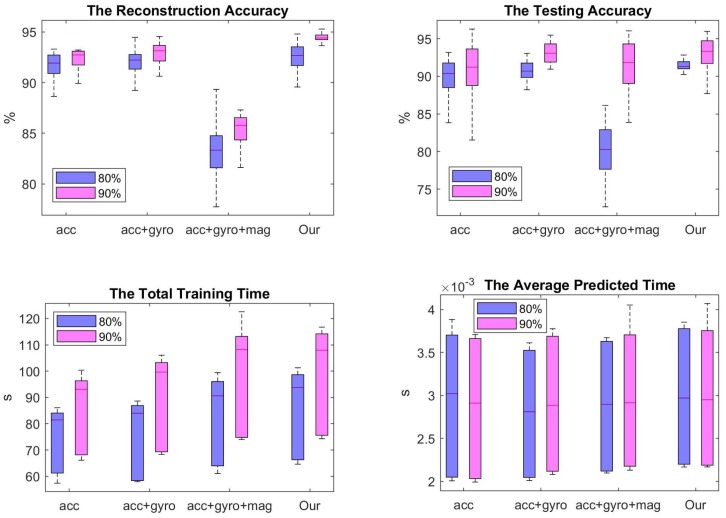
The comparison results among four types inputs: ’acc’ (${\tilde{s}}^{*}=s_{\alpha }$), ’acc+gyro’ (${\tilde{s}}^{*}=\left\{s_{\alpha },s_{\beta }\right\}$), ’acc+gyro+mag’ (${\tilde{s}}^{*}=\left\{s_{\alpha },s_{\beta },s_{\gamma }\right\}$), and ’Our’ (${\tilde{s}}^{*}=\left\{s_{\alpha^{1}},s_{\alpha^{2}},s_{\beta^{1}},s_{\delta_{Y,Z}}\right\}$). The reconstruction and testing accuracy are computed on the whole training and testing dataset, respectively. The total training time means the cost time for building the proposed DCNN classier. The average predicted time is to display the computational time to output an activity among these four kinds of inputs.

To evaluate the claims of the merits of the proposed FR-DCNN model in Section 1, we design several experiments to compare with the state-of-the-art DL algorithms and using different inputs. The experiments are implemented and evaluated these methods in MATLAB 2018b on a Windows PC with the hardware platform based on Intel(R) i7 Core 2.80 GHz CPU and 16.0 GB RAM. The first experiment is implemented to compare the classification accuracy, training time, and online recognition time among four types of inputs. It is also to evaluate the effectiveness of the described signal processing algorithms and signal selection module described in Section 2 (Figure 3). Due to these designing aims to enhance classification precision and avoid the time-consuming of training the DCNN classifier, the four picture drown by the comparison results are displayed in Figure 5.

The label ’acc’ on the x-axial means that it adopts the tri-axis acceleration signals as the inputs, namely ${\tilde{s}}^{*}=s_{\alpha }$. The ’acc+gyro’ means that the DL classifier use both 3-axis acceleration and 3D angular velocity as the inputs, namely ${\tilde{s}}^{*}=\left\{s_{\alpha },s_{\beta }\right\}$. The ’acc+gyro+mag’ means it adopts all of the raw signals collected from the three original IMU sensors, namely ${\tilde{s}}^{*}=\left\{s_{\alpha },s_{\beta },s_{\gamma }\right\}$. ’Our’ method means that it uses the 9D inputs selected from 3 to build the DCNN classifier, i.e., ${\tilde{s}}^{*}=\left\{s_{\alpha^{1}},s_{\alpha^{2}},s_{\beta^{1}},s_{\delta_{Y,Z}}\right\}$. We adopt the proposed DCNN structure to build the classifier. The results in blue boxes choose the first 80% datasets for training the DCNN classifier and the last 20% datasets for testing (called 80–20%), while the results of rad boxes are acquired by adopting 90% datasets for training the classifier and the last 10% for testing (called 90–10%). To save the experimental time, we remove 50% segments from each dataset with the same label for obtaining the training datasets, namely, ζ=0.5.

By observing the reconstruction accuracy shown in the top left picture 5, adopting all of the three IMU sensors as the inputs will decrease the recognition rate. Dut to the training datasets are compressed 50%, it cannot get 100% accuracy. Our method obtain the highest classification accuracy on the training datasets than the other kinds of inputs. Our method acquires up to 95% accuracy for reconstructing the whole training dataset. Specially, by testing on second experiment (90% training, 10% testing), our inputs ${\tilde{s}}^{*}=\left\{s_{\alpha^{1}},s_{\alpha^{2}},s_{\beta^{1}},s_{\delta_{Y,Z}}\right\}$ is the most robust method for recognizing the training data. By comparing the results on the testing data, the ’acc+gyro+mag’ inputs acquires the lowest accuracy in the 80–20% experiment. Even though it gets a good performance in the 90–10% experiment, it is not a robust method due to the computed large standard deviation. The length of boxes represents the robust of the method. Our method obtains a larger average accuracy 94% than the other methods in the 90–10% experiment. Specially, our method show the best robust in the 80–20% experiment. Although the classification accuracy obtained by ’acc+gyro’ inputs in 90–10% experiment is higher than that acquired in 80–20% experiment, it is a little bit lower than the accuracy obtained by ’our’ inputs. And the standard deviation of them are quit close to each other. The top two pictures in Figure 5 show the computed accuracy on ’acc+gyro+mag’ inputs is so much lower than that obtained from other inputs. Because the magnetometer is easy to be effected by various noises, which will affect the classification accuracy. However, this negative influence can be removed by the proposed FR-DCNN method. The orientation signals combine all of the three initial signals and we only use the data of *Y*-axis and *Z*-axis. The left bottom picture in Figure 5 display the training time for building a DCNN classifier. Even if adopting our method will cost a longer time (average 88s) than using the inputs ’acc+gyro’, our inputs ${\tilde{s}}^{*}=\left\{s_{\alpha^{1}},s_{\alpha^{2}},s_{\beta^{1}},s_{\delta_{Y,Z}}\right\}$ can obtain the robust results. The average prediction time (shown in the right bottom in Figure 5) of the four kinds of inputs are close to each other (0.0029 s), even if the online testing time obtained from ’acc+gyro’ inputs is lower than that from ’our’ inputs. Because only 0.0001 s difference cannot affect too much to the prediction speed. The most important aim of our work is to find a more strong algorithm to reduce the effect from the inputs, overfitting and underfitting. Meanwhile, by comparing the average prediction time in Figure 5, the dimension of the inputs has not affect the online recognition time. Hence, the proposed FR-DCNN algorithm provides a better method for increasing the classification accuracy and enhancing the robust of a classifier.

**Figure 6:**
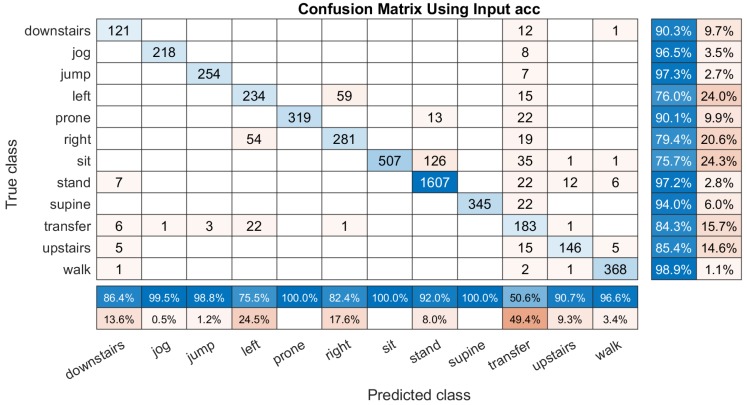
An example of the confusion matrix computed based on the input ’acc’ (${\tilde{s}}^{*}=s_{\alpha }$). It have a column summary matrix and a row summary matrix to display the accuracy and the errors of each activity.

**Figure 7:**
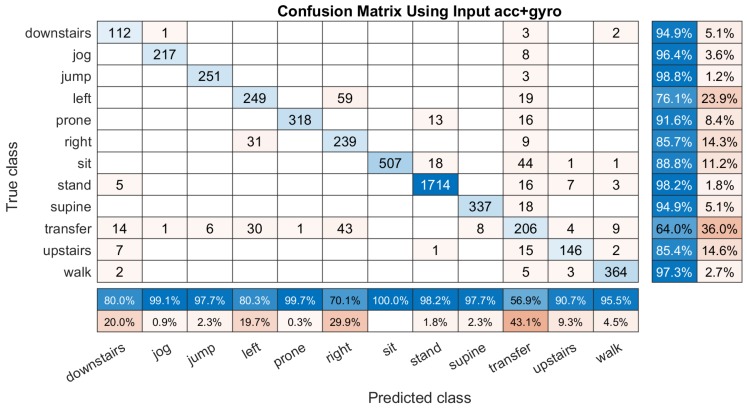
An example of the confusion matrix computed based on the input ’acc+gyro’ (${\tilde{s}}^{*}=\left\{s_{\alpha },s_{\beta }\right\}$). It have a column summary and row summary to display the accuracy and the errors of each activity.

**Figure 8:**
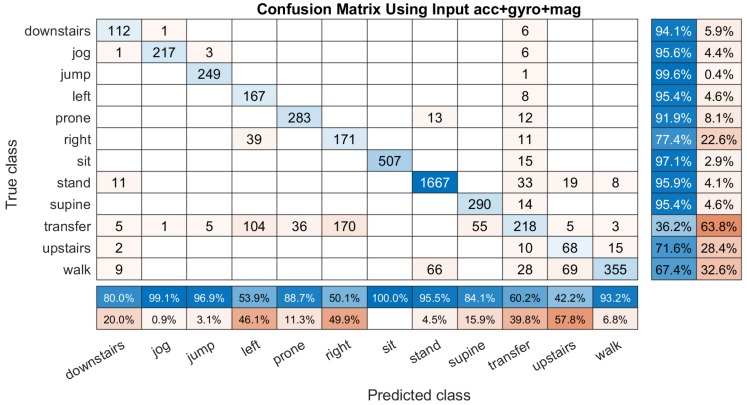
An example of the confusion matrix computed based on the input ’acc+gyro+mag’ (${\tilde{s}}^{*}=\left\{s_{\alpha },s_{\beta },s_{\gamma }\right\}$). It have a column summary matrix and a row summary matrix to display the accuracy and the errors of each activity.

**Figure 9:**
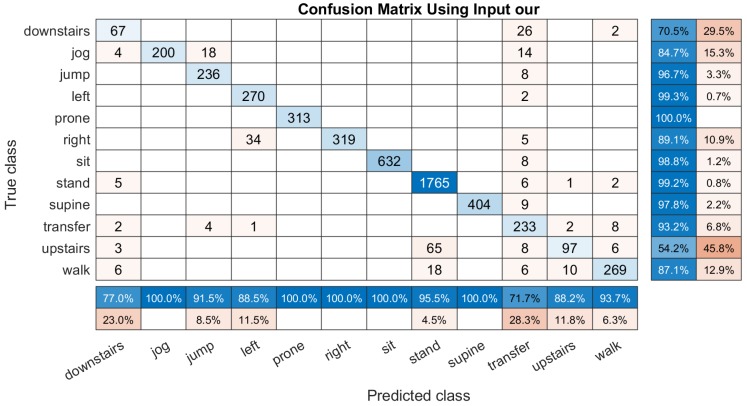
An example of the confusion matrix computed based on our input ${\tilde{s}}^{*}=\left\{s_{\alpha^{1}},s_{\alpha^{2}},s_{\beta^{1}},s_{\delta_{Y,Z}}\right\}$. It have a column summary and row summary to display the accuracy and the errors of each activity.

For more in-depth verification the efficiency of input dimensions, we draw the four confusion matrix examples of the mentioned four types of inputs from Figure 6, Figure 7, Figure 8 and Figure 9. All of these experiments are tested on 5088 samples. Most of the activities can be identified only using the tri-axis accelerometer shown in Figure 6. The total classification accuracy is 90.07%. However, using ’acc’ inputs it is difficult to identify the transition from other dynamical activities. It has 49.4% error, and the lying to left-side position is recognized by the right-side and ’transferred’.

However, adopting only two IMU sensors, i.e., 3D accelerometer and tri-axis gyroscope, can get better performance for identifying the 12 activities. As shown in Figure 7, the total classification accuracy improved to 91.59% and the recognition rate of ’transfer’ is better than using ’acc’ in Figure 6, and the established DCNN classifier has not sacrificed too much of the accuracy for identifying the four lying positions.

After adding both gyroscope and magnetometer information, the miss classification rate does not acquire improvement, even worse. Figure 8 shows the obtained confusion matrix based on ’acc+gyro+mag’ inputs and the total classification accuracy is 84.59% which is so much lower than the previous two experiments. Almost all of the activities are recognized into wrong classes. Specially, the ’upstairs’ lying to the left and right sides can not be identified well. Meanwhile, most of the transition activities are miss classified. Although the classification accuracy (60.2%) of ’transfer’ gets better than that only use ’acc’ as the inputs (50.6%), using all of the three IMU sensors (’acc+gyro+mag’) is the worst method for building the DCNN classifier.

By comparing with all of the above three types of inputs, our method to select the inputs ${\tilde{s}}^{*}=\left\{s_{\alpha^{1}},s_{\alpha^{2}},s_{\beta^{1}},s_{\delta_{Y,Z}}\right\}$ for building the DCNN classifier is proven to obtain a higher classification accuracy. Figure 9 shows the computed confusion matrix by the DCNN classifier built on ’our’ inputs. The total classification accuracy is 96.34%. It can be seen that most of the ’transfer’ activities are classified into the correct class with 72.9% accuracy in the raw-summary and 93.5% accuracy the column summary. The only worst classification happens on identifying ’upstairs’; it gets 53.1% error in the column summary because some of them are recognized into the ’stand’ class. Specially, our method get 100% recognition rate for recognizing lying supine, prone, right, sitting and jogging, which is evaluated so much better than the other three methods.

Table 1 compares the HAR performance between the designed DCNN classifier and other popular DL classifier, i.e., LSTM, Bi-LSTM and the other two DCNN model with 3 and 5 CNN modules, by building the DCNN classifier on 50% (ζ=0.5) compressed training datasets. It uses the 90% data (collected from 18 subjects) for training and the last 10% data (sampled from two subjects) for testing. To enhance the persuasive capability of this comparison experiment, all of the algorithms are tested 20 times to compute the average and standard deviation of each results set. The proposed FR-DCNN have four convolutional modules including 2D CNN layers, a batch normalization (BN) layer and a Rectified Linear Unit (ReLU). In these experiment, we compare the FR-DCNN method with three and five convolutional modules, called 3-Module DCNN and 5-Module DCNN. The replacement optimization algorithm is the adaptive moment estimation optimizer (adam). The optimization parameters of DCNN model are set as follows. The learn rate is 0.001 with 0.1 drop factor and 100 drop period. The LSTM model consists of one LSTM layer with 50 neurons and 50 size of minibatch, a fully connected layer, a softmax layer and a classification layer. Similarly, the Bi-LSTM model adopts the same layers as LSTM model with the 50 nodes Bi-LSTM layer. Both LSTM and Bi-LSTM models use the same learning rate, drop factor and drop period with DCNN models. By comparing the classification accuracy calculated on the whole training dataset, our FR-DCNN method get the highest reconstruction rate (94.09%) than all of the other methods. Although the Bi-LSTM classifier is the most robust method because it obtains the lowest standard deviation (0.0065), out method also get a close value with Bi-LSTM (0.0070). Hence, FR-DCNN method is still the best algorithm for reconstruct the training dataset. By comparing the accuracy computed on the testing dataset, although our FR-DCNN method obtain a little bit lower average accuracy (94.18%) than the LSTM and Bi-LSTM methods (95.39% and 95.35%, respectively), the FR-DCNN method only spend 88s to build the DCNN classifier. That is too much less than the LSTM, Bi-LSTM, and 5-Module DCNN algorithms. Meanwhile, our FR-DCNN method is one of the fast classifier to output a result (activity) on testing data. It only need 0.0022s (average time) to predict the activity on a signal segment, while the Bi-LSTM needs 0.0142 s.

As the data compression module is designed to remove similar training segments, which might cause slow building of the DL classifier, the compressed training dataset is expected to leave the most useful information for establishing the DCNN classifier. Hence, the difference of the comparative results calculated based on the whole training dataset (90%) among FR-DCNN, two kinds of DCNN, LSTM, and Bi-LSTM should be similar to the results acquired in Table 2. Although the classification accuracy obtained on the training dataset is higher than the results computed on the compressed training set (specially, the FR-DCNN method acquires 96.88% accuracy), they cost more time to train the model. For example, the FR-DCNN model need 179.38 s to build the classifier, which is the twice of that obtained on the compressed dataset.

**Figure 10:**
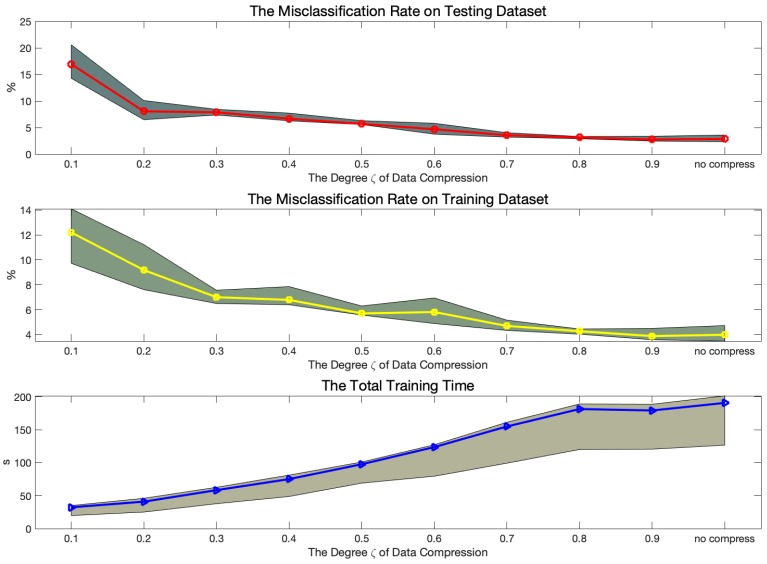
The performance trend with the increasing degree of data compression ζ. The top two pictures show the error rates on the testing and training datasets. The bottom picture is the whole training time. The shaded parts represent the Quartile range from 0.25 to 0.75.

Figure 10 displays the errors and training time trend obtained on the last 10% testing dataset. Since the compression degree ζ is an significant decisive factor for maintaining the consistency between the compressed and without compressed training dataset, we set the value ζ from 0.1 to 0.9 for comparing the errors computed on the whole training and testing dataset and the time of building the DCNN classifier. By observing the misclassification rate in the top two pictures shown in Figure 10, the errors decrease along with the increase of value ζ. To avoid the defects of NN such as overfitting and underfitting, all of the models are tested 20 times and the middle line of each picture is the average values. The shaded parts represent the Quartile range (0.25–0.75) of the results of each experiment. The trend of total training time grows when the compression degree is increase. Despite the structure cost about 90 s to build the DCNN classifier on the 50% compressed dataset, it is the best degree for both fast computation and high accuracy classification.

**Figure 11:**
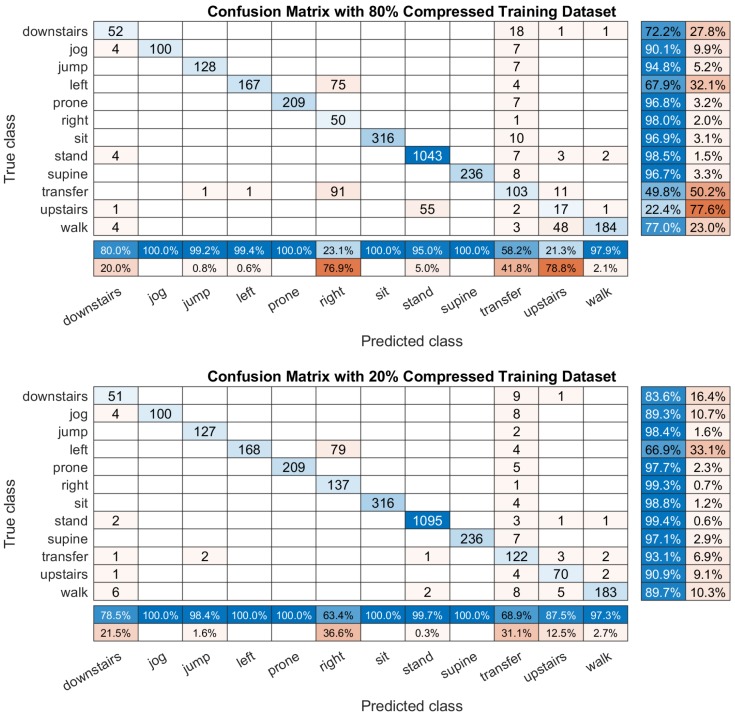
An example of the confusion matrix computed based on 80% and 20% compression training dataset. The top matrix is the prediction results computed by the DCNN classifier building on the 80% compressed dataset. The bottom picture is the results by the same classifier established on the 20% compressed training dataset. All of the matrix have a row summary to display the accuracy and the errors of each activity.

Figure 11 shows two examples of the confusion matrix computed by the DCNN classifier which is built on two different training datasets. The first one is compressed 80% (ζ=0.2), while the second one is compressed 20% (ζ=0.8). The whole classification accuracy is 87.36% and 94.37%, respectively. By observing the accuracy for identifying each activity in the row summary matrix on the right, the 80% compression training dataset classify too may ’downstairs’ to the ’walk’, and it lost the recognition ability for identifying the transition. However, the recognition rate gain improvement on the 20% compressed training dataset. It not only solve the problem of identifying ’transition’ and ’upstairs’ activities, but also increase the whole classification accuracy.

## 4. Conclusions

In this paper, we proposed a fast and robust deep convolutional neural network (FR-DCNN) architecture to perform complex activity recognition from smartphone sensors. The new framework decreases the computational time to training a DCNN classifier through the designed data compressing module to remove the similar components in the same labeled dataset. By comparing the training time and classification accuracy along with the different compression degrees ζ, it is found that deleting 50% of the training data not only saves half of the training time, but also keeps the high precision of predicting the 12 activities. The FR-DCNN method focuses on complex HAR. Except recognizing the typical activities in the previously published contributions, such as jumping, jogging, and walking, the FR-DCNN algorithm can identify four types of lying positions (left and right sides, lying supine and prone) and a series of transitions. All of these activities are the current challenges of HAR area. Furthermore, we design a series of measures in the FR-DCNN model to enhance classification accuracy. In this study, it conducts extensive research to explore the power of the triaxial accelerometer and gyroscope in activity recognition. The signal selection module chooses the most useful signals obtained from the signal processing module. The used 9D inputs for recognizing the 12 activities outperformed the results computed based on other inputs. Especially, the experimental results show that the used 9D inputs can provide more discriminant information than adopting the fusion of accelerometer and gyroscope data contributes to obtaining better classification performance. Besides, the designed DCNN model outperformed the results obtained from other DL algorithms, like LSTM, Bi-LSTM, and DCNN models with 3 and 5 CNN modules in the collected training dataset by ~1% on average. Although the DCNN method is not the best algorithm for predicting the activities on the testing dataset (actually, the LSTM get about 1% average better than DCNN), it only needs less than half time of LSTM to train the classifier and cost 25% time of LSTM to predict an output.

For future work, we will investigate a transfer learning approach to conduct further research in the following lines. First, human activity is an open mind and complex issue, which can not be regarded as a simple multiple classification problem. Therefore, establishing a classifier on the less labeled dataset for implementing on the extensive unknown data becomes the most popular challenge. Second, exploring other active and fast clustering or classification algorithms and comparing them with the proposed one in this study remains a topic for future research. Third, perceiving the state of an individual and development of human-centric applications are helped by the accurate activity recognition. Therefore, developing an assisted living system in order to understand users behavior patterns will promote healthcare in a home setting.

## Figures and Tables

**Table 1:** The comparison performance on the compressed training dataset (ζ=0.5) among FR-DCNN, DCNN, LSTM, and Bi-LSTM.

Algorithms	Accuracy		Computational Time	
	Train	Test	Train	Test
FR-DCNN	94.09% ± 0.0070	94.18% ± 0.0170	88.00 s ± 15.57	0.0028 s ± 0.0007
3 Modules DCNN	93.92% ± 0.0075	93.04% ± 0.0170	71.75 s ± 13.32	0.0022 s ± 0.0006
5 Modules DCNN	93.23% ± 0.0317	93.06% ± 0.0308	118.32 s ± 18.32	0.0036 s ± 0.0009
LSTM	93.59% ± 0.0103	95.39% ± 0.0178	226.29 s ± 3.92	0.0118 s ± 0.0004
Bi-LSTM	93.65% ± 0.0065	95.35% ± 0.0157	266.28 s ± 6.61	0.0142 s ± 0.0004

**Table 2:** The comparison performance on the whole training dataset (90%) among FR-DCNN, DCNNs, LSTM, and Bi-LSTM.

Algorithms	Accuracy		Computational Time	
	Train	Test	Train	Test
FR-DCNN	96.88% ± 0.0050	95.27% ± 0.0160	179.38 s ± 36.41	0.0029 s ± 0.0008
3 Modules DCNN	96.06% ± 0.0055	93.61% ± 0.0163	135.28 s ± 26.45	0.0022 s ± 0.0006
5 Modules DCNN	95.60% ± 0.0090	94.26% ± 0.0166	226.24 s ± 36.62	0.0038 s ± 0.0008
LSTM	94.55% ± 0.0088	96.43% ± 0.0077	406.28 s ± 4.53	0.0115 s ± 0.0001
Bi-LSTM	94.52% ± 0.0080	96.42% ± 0.0107	515.76 s ± 28.35	0.0143 s ± 0.0007
